# Tryptophan metabolites exert potential therapeutic activity in graves’ orbitopathy by ameliorating orbital fibroblasts inflammation and proliferation

**DOI:** 10.1007/s40618-025-02593-6

**Published:** 2025-05-27

**Authors:** Weili Yang, Xinyu Xu, Rongrong Xie, Jiaqi Lin, Zhijia Hou, Zhong Xin, Xi Cao, Tingting Shi

**Affiliations:** 1 Beijing Diabetes Institute, Beijing Key Laboratory of Diabetes Research and Care, Department of Endocrinology, Beijing Tongren Hospital, Capital Medical University, Beijing, 100730 China; 2 Department of Endocrinology, Beijing Tongren Hospital, Capital Medical University, Beijing, 100176 China; 3 Beijing Tongren Hospital, Beijing Tongren Eye Center, Capital Medical University, Beijing, 100176 China; 4 Beijing Ophthalmology and Visual Science Key Lab, Beijing, 100176 China

**Keywords:** Graves’ orbitopathy, Gut Microbiome, Orbital fibroblasts, Inflammation, Proliferation, Tryptophan metabolites

## Abstract

**Purpose:**

Graves’ orbitopathy (GO) is a sight-threatening organ-specific autoimmune disease with complicated pathogenesis. Gut microbiota-derived tryptophan (Trp) metabolites play important roles in immune-related diseases, but their role in GO remains unknown.

**Methods:**

Trp metabolism-associated gut flora was analyzed by 16 S sequencing in GO patients and controls. Serum metabolomics profiling was performed to assess Trp metabolic pathway. Trp metabolites levels were measured by ELISA in 401 serum samples from a case-control study, and their effects on inflammation and proliferation in orbital fibroblasts were evaluated in vitro.

**Results:**

Trp metabolism-associated gut flora, including phylum Firmicutes and genus Anaerostipes, were significantly down-regulated in GO patients. Serum metabolomics revealed significant enrichment of Trp metabolic pathway in both GO and Graves’ disease (GD) groups. Serum levels of indolepropionic acid (IPA), indole-3-lactate (ILA), and indoleacetic acid (IAA) were significantly decreased in both GD and GO patients compared to controls, with IAA levels further reduced in GO compared to GD patients. Notably, active GO patients had significantly lower IAA levels compared to inactive ones. Moreover, the levels of IAA were negatively correlated with clinical activity score and serum thyrotropin receptor antibody (TRAb) in GO patients. In vitro, IPA, ILA, and IAA mitigated TNFα-induced inflammation and proliferation in orbital fibroblasts by suppressing the Akt signaling pathway.

**Conclusion:**

Trp metabolites IAA maybe a novel biomarker for GO progression. And IPA, ILA and IAA may play a protective role in GO by regulating inflammation and proliferation in orbital fibroblasts, suggesting their potential as therapeutic targets for GO treatment.

**Supplementary Information:**

The online version contains supplementary material available at 10.1007/s40618-025-02593-6.

## Introduction

Graves’ orbitopathy (GO), also known as thyroid-associated ophthalmopathy and thyroid eye disease, is primarily a thyroid-related organ-specific autoimmune disease [1]. It predominantly occurs in patients with hyperthyroidism and can also be observed in cases of euthyroidism and hypothyroidism [2]. The characteristic manifestations, including eyelid contracture, proptosis and vision loss, impose a significant burden on society and families, seriously impacting individuals’ health and quality of life [3]. However, the underlying mechanisms of GO are complex, including the trigger by autoimmune reactions against thyroid stimulating hormone receptor (TSHR) and Insulin-like growth factor-1 receptor (IGF-1R), necessitating further in-depth investigation. Management of GO usually requires a multidisciplinary approach. Treatment of active moderate-to-severe GO mainly relies on intravenous glucocorticoids or in combination with other therapies including mycophenolate or orbital radiotherapy. Excitingly, novel biological agents including Teprotumumab (target IGF-1 receptor), Rituximab (target CD20-positive B cells), and Tocilizumab (target IL-6 receptor) have achieved encouraging results [4]. In clinic, early identification of severe and active GO is important, appropriate treatments will be selected according to the phase and degree of orbital involvement. So, exploring sensitive biomarkers for GO progression and then revealing new potential treatment targets is urgent.

Nowadays, the microbiota-gut-thyroid axis has been proposed to reveal the crosstalk between the thyroid and intestinal microbia [5, 6]. Our previous study evidenced alterations in the bacterial composition and metabolite profiles in GO patients [7]. GO patients demonstrated changes in gut microbiota including reduced bacterial diversity and uneven microbial composition. Gut microbiota also plays an important role in tryptophan (Trp) metabolism, which is involved in gut immune homeostasis and, consequently, immune diseases [8, 9]. The metabolism of Trp mainly involves three metabolic pathways: kynurenine, 5-hydroxytryptamine, and indole pathways. Kynurenine pathway primarily takes place in the liver by Trp-2,3-dioxygenase and indoleamine-2,3-dioxygenase, 5-hydroxytryptamine pathway takes place in both the gut and brain by Trp hydroxylase, while indole pathway takes place in the gut and the gut microbiota directly metabolizes Trp to indole, indole derivatives, tryptamine, and skatole [10]. Indole derivatives, such as indole-3-lactate (ILA), indole-3-acrylate, indole-3-propionate (IPA), indole-3-aldehyde, indoleacetic acid (IAA) and indole-3-acetaldehyde can be produced by intestinal microorganisms through direct Trp transformation [11]. For example, *Clostridia* converts Trp into ILA and IPA [12]. It has been demonstrated that Trp metabolites can maintain the function and homeostasis of immune cells [13]. And Trp metabolites play important role in controlling the differentiation and functions of immune cells including T cells [14], B cells [15], and lymphocytes [16]. However, whether changes in gut microbiota in GO patients affect Trp metabolism and whether Trp metabolites have an effect on GO remain unknown.

The pathogenesis of GO primarily involves the activation of orbital fibroblasts (OFs), immune cell infiltration, and hyaluronan deposition resulting in orbital tissue expansion and muscle hypertrophy [17, 18]. OFs, which can differentiate into myofibroblasts and produce extracellular matrix components when activated, are considered to be critical in inflammation and tissue remodeling in GO [19]. To date, there is no report that has investigated the effects of Trp metabolites on OFs in GO. First, this study intended to identify changes in Trp-metabolizing gut microbes and microbiota-derived Trp metabolites in GO patients by analyzing intestinal microbiota and serum metabolomics data respectively. We found that Trp metabolism-associated microbiota were altered in GO patients and the Trp corresponding enrichment pathways were significantly changed, with ILA, IPA and IAA standing out as particularly noteworthy. Second, we investigated the serum levels of IPA, ILA and IAA in GO patients compared with those in Graves’ disease (GD) patients and healthy volunteers using enzyme-linked immunosorbent assays (ELISA). And lastly, we investigated the effects of IPA, ILA and IAA on the proliferation and inflammation of primary cultured human OFs in vitro, aiming to clarify the potential mechanisms. All in all, we combined clinical sample detection with in vitro cell experiments, aiming to explore whether Trp metabolites can serve as novel biomarkers for GO progression and new potential therapeutic targets for GO treatment.

## Materials and methods

### Ethical approval statement

The current study was approved by the Institutional Review Board of Beijing Tongren Hospital, Capital Medical University (ethical no. TRECKY2016-003, TREC2020-XJS02). Written informed consent was obtained from all the patients and volunteers.

### Study design

For the part of clinical sample detection in the study, the clinical design was a case-control study. Our primary objective for the clinical study was identifying the difference in levels of Trp metabolites among GD patients, GO patients and healthy volunteers. The secondary objective for the clinical study was exploring the correlation between Trp metabolites and clinical indexes including TRAb and CAS.

### Sample size calculation

According to the results of the preliminary pilot experiment, it was found the serum levels of IAA were 38.14 ± 6.54 (ng/mL), 37.95 ± 9.28 (ng/mL), and 33.09 ± 10.95 (ng/mL) for controls, GD patients and GO patients, respectively. With an alpha level set at 0.05, a test power of 90%, and a two-sided test design, the sample size calculation was conducted in parallel for the measurement data of the three groups based on the equal allocation ratio. As calculated by using the One-Way Analysis of Variance F-tests module of PASS 15.0 software, the sample size was 94 participants for each group. In the current study, the actual data collected included 100 participants in the control group, 145 patients in the GD group, and 156 patients in the GO group.

## Sample collection

For the part of clinical sample detection in the study, we enrolled healthy volunteers and all patients from the Department of Endocrinology in Beijing Tongren Hospital who met the inclusion and exclusion criteria mentioned below from January 2020 to March 2022. Those included 100 samples from healthy volunteers (Control), 145 samples from GD patients, 156 samples from GO patients. GD diagnosis was made clinically with diffuse goiter, elevations in serum thyroxine (T4) and a suppressed thyroid stimulating hormone (TSH) levels, combined with TSH-receptor (TSH-R) antibody (TRAb) testing, in accordance with the 2016 American Thyroid Association Guidelines [20]. The diagnosis of GO was established according to the European Group on Graves Orbitopathy (EUGOGO) guidelines [1, 21]. The activity and severity of GO were established according to the EUGOGO Guidelines. The active GO was defined by a clinical activity score (CAS) ≥ 3, and the severe GO was defined by NOSPECS score ≥ IV. Orbit CT or MRI was used to exclude any orbital space-occupying disease. All patients with hyperthyroidism were treated exclusively with antithyroid drugs (Methimazole, Merck Company, Germany). We collected the clinical and demographic data including gender, age, thyroid function, thyroid autoantibodies and case history. The exclusion criteria for the study included age < 18 or > 65 years, therapy of probiotics or antibiotics in the past 1 month, use of hormonal medication or Chinese herbal medicine, and history of chronic diarrhea or constipation, systemic disease (diabetes, stroke, heart disease, renal or hepatic dysfunction and cancer), history of gastrointestinal tract surgery, pregnancy and lactation, pure vegetarian, alcohol or substance addiction. All the enrolled subjects were Han nationality. Serum of the enrolled subjects were collected and stored at −80℃ until further ELISA examination.

## ELISA assay of serum Trp metabolites

Serum samples were collected and stored at −80℃. The samples were analyzed using commercially available ELISA following the manufacturer’s instructions. Levels of IAA were measured using the Human IAA (Cat. No. CEA737Ge) ELISA kits (Cloud-Clone Corp, Houston, Texas, USA). IPA and ILA were measured using the Human IPA (Cat. No. MM-61644H1) and Human ILA (Cat. No. MM-61694H1) ELISA kit (Jiangsu Meimian Industrial Co., Ltd.). The kits were designed for use with human serum or plasma samples and showed no cross-reactions.

## 16 S sequencing

16 S sequencing was conducted as part of our previous research [7]. Briefly, 33 patients with severe and active GO and 32 healthy volunteers (Control) were enrolled from the Out-patient Department of Endocrinology, Beijing Tongren Hospital, Capital Medical University between March 2017 and March 2018. Human fecal samples (2–3 g) were collected in sterile stool cups and stored at −80℃ until further processing. The microbial DNA was extracted by the E.Z.N.A. Stool DNA kit (Omega inc., USA) and the total DNA was eluted in 50 µL of elution buffer. The extracted DNA was stored at −80℃ until measurement.

### Serum metabolomics profile analysis

Serum metabolomics profile analysis was conducted as part of our previous research. Briefly, 16 healthy volunteers (Control), 16 GD patients and 31 severe and active GO patients were enrolled from the Out-patient Department of Endocrinology, Beijing Tongren Hospital, Capital Medical University between March 2017 and March 2018. Serum of the enrolled subjects were collected and stored at −80℃ until further metabolomics profile analysis. Metabolites with significant differences were selected based on the projected variable importance (VIP) scores (obtained from the orthogonal least squares discriminant analysis model) and the p-value of the Wilcoxon test (VIP threshold > 1, *p* < 0.05). Partial Least Squares Discrimination Analysis (PLS-DA) was used to evaluate the cross-validation of the samples. The biochemical pathways of the differential metabolites were identified using the Kyoto Encyclopedia of Genes and Genomes (KEGG) database and classified by their involvement in the pathways. Enrichment analysis was performed based on the presence of metabolites in functional nodes. Stats’ Python package was used to test the statistical significance of the enriched pathways identified by Fisher’s exact test.

## Cell culture and reagents

Orbital connective tissue explants were obtained from patients undergoing orbital decompression surgery for severe GO (*n* = 4) at Beijing Tongren Hospital, Capital Medical University. Control orbital tissues (*n* = 5) were collected from individuals without thyroid or inflammatory disease or tumors who underwent enucleation after injuries. This study was approved by the Ethics Committee of Beijing Tongren Hospital, Capital Medical University. The human OFs cultures were initiated from orbital connective tissue explants as described in the literature [22]. Briefly, orbital connective tissue explants were washed three times with phosphate-buffered saline (PBS) and cut into small pieces immediately after being obtained and the tissues were placed directly in culture dishes. Afte adherence, explants were immersed in DMEM/F12 medium (Sigma-Aldrich, St. Louis, MO, USA) containing 20% fetal bovine serum (FBS; Gibco, Carlsbad, CA) and 1% penicillin/streptomycin (Thermo Fisher Scientific). The cultures were maintained at 37 °C in a humidified atmosphere of 95% air and 5% CO_2_. OFs generally migrate from explants within approximately 4 days and reached confluence in about 10 days. Then the cells were passaged with 0.25% trypsin/EDTA (Gibco Laboratories, New York, USA). After centrifugation at 300 g for 5 min and room temperature, the supernatant was discarded, and the cell pellet was resuspended in 10 mL of DMEM/F12 medium and then filtered through a 70-mm cell mesh to establish a cell line. After the first cell passage, the OFs were maintained in DMEM/F12 medium containing 10% FBS and 1% penicillin/streptomycin with the culture medium changed every 2 to 3 days. The ranges from third to sixth cell passages were used in the experiments. 3-Indoleacetic acid (IAA, Cat. No. GC33436), 3-Indolepropionic acid (IPA, Cat. No. GC31290) and Indolelactic acid (ILA, Cat. No. GC33659) were purchased from GlpBio (California, USA). Human recombinant protein tumor necrosis factor alpha (rHu TNFα) was purchased from Sangon Biotech (Shanghai, China).

## Cell proliferation experiment

The proliferation activity of the OFs was determined by Cell Counting Kit-8 assay (CCK-8; TransGen, Beijing, China) as previously described [23]. Briefly, OFs were seeded into a 96-well plate, and after reaching 80% confluence, the cells were treated with different concentrations of the 3 Trp metabolites or the equivalent amount of solvent (DMSO) for 24 hours (h). The working concentrations of these Trp metabolites were as below: IPA (10, 50 and 100 µM), ILA (0.1, 0.5 and 1 mM), IAA (25, 100 and 400 µM). Cells treated with the equivalent amount of solvent served as the control group. To evaluate the anti-proliferation effect of the IPA, ILA and IAA induced by rHu TNFα, OFs were treated with 20 ng/ml rHu TNFα or the equivalent amount of solvent, along with IPA (50 µM), ILA (0.5 mM) or IAA (100 µM) for 24 h. 0.5–1 h before the end of incubation period, the CCK-8 solution was added to each well of the 96-well plate at a final concentration of 10%, and gently mixed. After 0.5–1 h, absorbance (A) was measured at 450 nm with a reference wavelength of 630 nm. The OFs proliferation activity was showed as the relative cell viability which was determined using the following formula: Relative cell viability = (A450/A630 of an experimental group)/(A450/A630 of the control group). Stock solutions of all the three Trp metabolites were prepared in DMSO and diluted in the culture medium to obtain the desired final concentrations. The final DMSO concentration in the culture medium did not exceed 0.5% (v/v).

### Western blot assay

OFs were seeded into a 6-well plate, and after reaching 80% confluence, the cells were treated with 20 ng/ml rHu TNFα or the equivalent amount of solvent, along with IPA (50 µM), ILA (0.5 mM) or IAA (100 µM) or the equivalent amount of solvent at the same time for 24 h. Western blot assays were implemented in a standard manner as described previously [24–26]. Anti-TNFα (3707 S), Anti-pStat3 (9145 S), Anti-Stat3 (12640 S), Anti-Protein kinase B(Akt) (9272 S) and Anti-pAkt (4060 S) were purchased from Cell Signaling Technology. Anti-β-actin was purchased from sigma (A5316). Each band was quantified as image intensity and assessed by the ImageJ software (National Institutes of Health, Bethesda, MD, USA). β-actin was utilized for a normalization control in the experiments.

### Statistical analysis

Demographic data, clinical data and laboratory tests were analyzed using IBM SPSS 27.0 software and GraphPad Prism software, version 8.0.1. Normally distributed continuous variables were expressed as means and standard deviation (SD), while non-parametric variables were expressed as median (Q1, Q3). For comparisons between different groups, Student’s t test, one-way ANOVA and Kruskal-Walli’s test were used for two-group and multiple-group comparisons, respectively. *P* < 0.05 was considered statistically significant. For the differential impact analysis, Benjamini and Hochberg FDR (BH) correction for dependent multiple comparisons was used to correct for large number of analyses. All correlations between variables were determined by Spearman’s rank correlation.

## Results

### Trp metabolism associated intestinal flora altered in GO patients

**Fig. 1:**
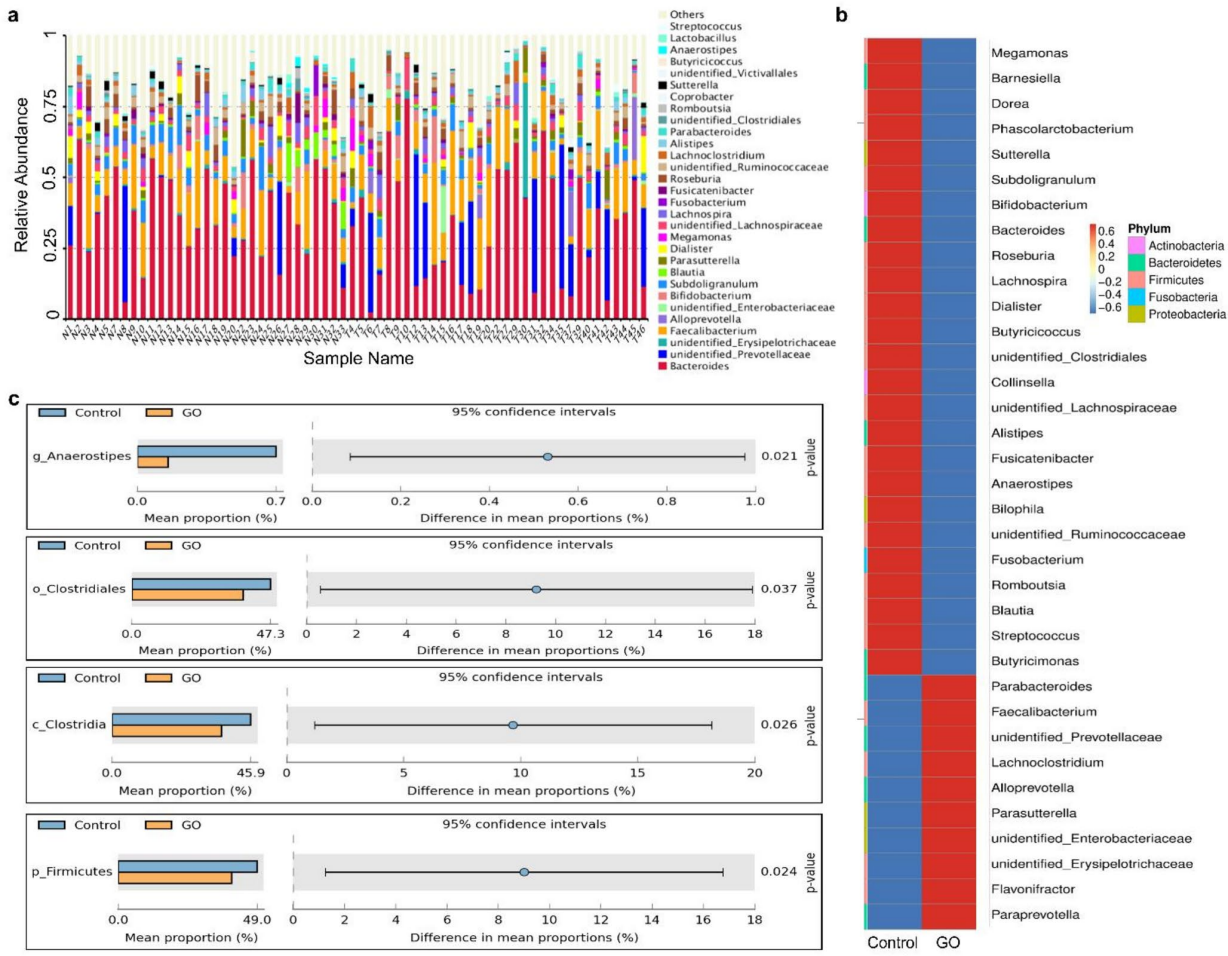
Trp metabolism associated intestinal flora altered in GO patients. **a** Histogram of species distribution for the top 30 gut flora at the genus level. **b** Taxa heatmap showing the abundances of 35 genera at the genus level.**c**, Disparities of bacterial taxa producing Trp metabolites at the genus, order, class, and phylum levels. Control: Healthy volunteers; GO: Graves’ orbitopathy

Our previous 16 S sequencing study showed significant differences in α diversity and β diversity indexes between fecal samples from GO patients and controls [7]. However, whether there are any changes in Trp-metabolizing gut microbes remain unexplored. In this study, the intestinal flora was identified and analyzed at the phylum (p), class (c), order (o), family (f), and genus (g) levels. The histogram of species distribution of the top 30 gut flora at the genus level shows that species were mainly distributed among B*acteroides*, *Prevotellaceae* and *Faecalibacterium* in two groups (Fig. 1a). Additionally, the taxa heatmap showed the abundances of 35 genera in GO patients were significantly different when compared to Control, which were belong to five phylums including *Actinobacteria*, *Bacteroidetes*, *Firmicutes*, *Fusobacteria*, and *Proteobacteria* (Fig. 1b). As we described in the introduction, several bacterial species are known to convert Trp into indole and its derivatives. In order to reveal the role of Trp metabolism in GO, we further analyzed changes in Trp metabolism-related flora in Control and GO patients at different levels. The results showed that at the genus level, abundance of *g_Anaerostipes*, which can convert Trp into ILA, in GO group were markedly decreased compared to Control; At the order level, abundance of *o_Clostridiales*, which can convert Trp into ILA, in GO group were markedly decreased compared to Control; At the class level, abundance of *c_Clostridia*, which can convert Trp into ILA, IPA and IAA, in GO group were markedly down-regulated compared to Control; At the phylum level, abundance of *p_Firmicutes*, which can convert Trp into ILA, in GO group were significantly down-regulated compared to Control (Fig. 1c). These results suggested that Trp metabolism-associated gut flora, including phylum *Firmicutes* and genus *Anaerostipes*, might be closely related with GO disease. And with the significant decline of Trp-metabolizing gut microbes, it is likely that their associated Trp metabolomics IAA, ILA and IPA were also significantly reduced, suggesting the occurrence of GO may be closely related to the metabolism of Trp in intestinal flora, with ILA, IPA and IAA standing out as particularly noteworthy.

### Alteration in serum metabolomics profile in GO patients

**Fig. 2:**
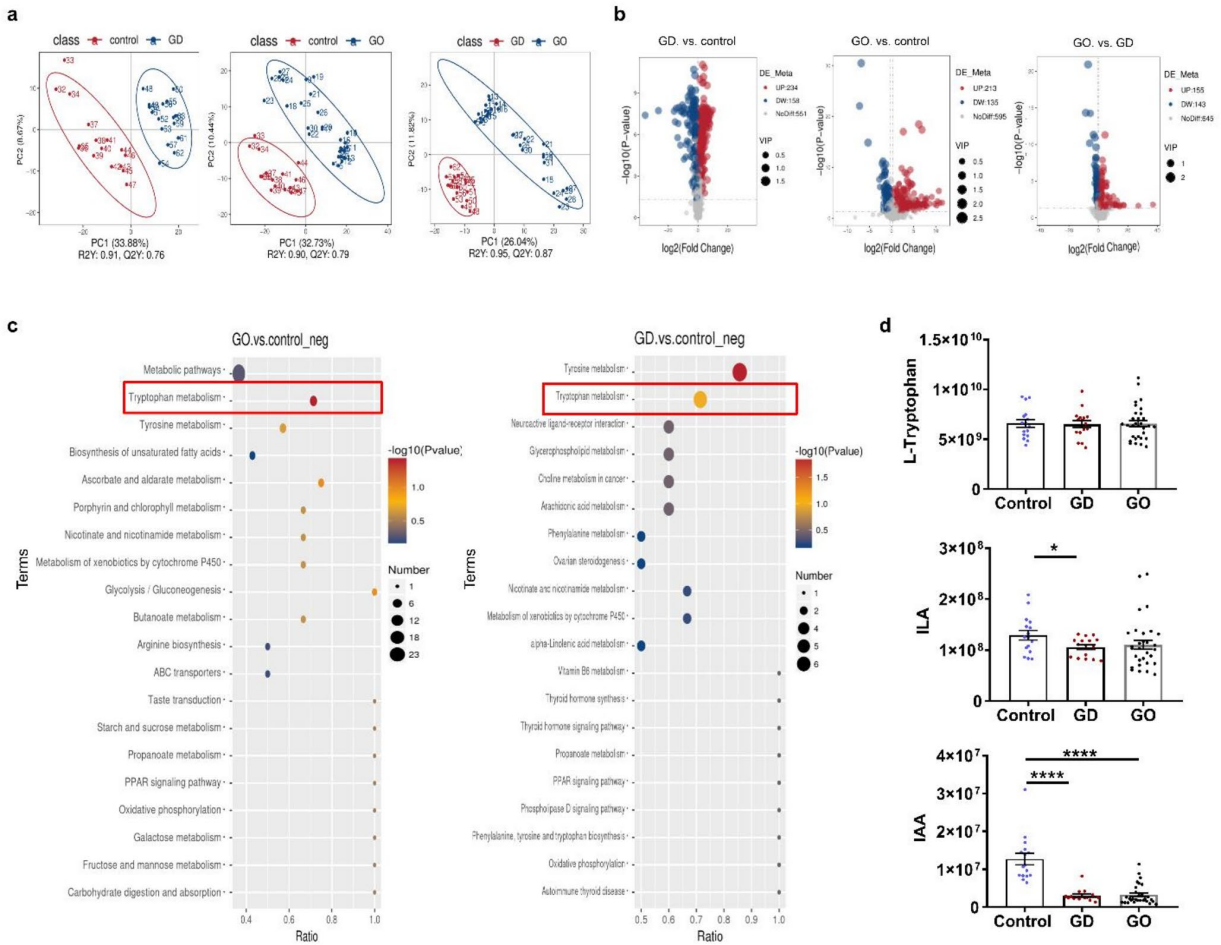
Alteration in serum metabolomics profile in GO patients. In the context of metabolomic research, comparative analyses were conducted on patient cohorts with GO and GD compared with Control. **a** Partial Least Squares Discrimination Analysis (PLS-DA) of GO, GD, and Control groups represented in positive ion mode. **b** Volcano plots depicting metabolite profiles in GO, GD patients compared with controls. Blue and red dots indicate the depletion and enrichment of metabolites, respectively. A dashed line denotes a critical p-value threshold of 0.05. **c** Bubble plots illustrating KEGG enrichment analysis, where bubble size corresponds to the number of enriched metabolites in a pathway, and color gradient indicates the degree of enrichment significance. GO and GD patient groups were individually compared with controls. **d** Differences in metabolite profiles among Control, GD and GO groups. *p < 0.05; ****P < 0.0001 vs. Control. Control: Healthy volunteers; GD: Graves’ disease; GO: Graves’ orbitopathy; ILA: indole-3-lactate; IAA: indoleacetic acid

To further elucidate the change of Trp metabolomics in GO patients compared with GD patients and controls, we performed serum metabolomics profiling in three groups. A total of 2182 metabolites were quantified in this experiment. Supervised partial least square discriminant analysis (PLS-DA) revealed a clear separation between the control and GD groups (R2Y = 0.91, Q2Y = 0.76), the control and GO groups (R2Y = 0.90, Q2Y = 0.79), and the GD and GO groups (R2Y = 0.95, Q2Y = 0.87), the values, all close to 1.0, indicated all the 3 models were stable and demonstrated predictive reliability (Fig. 2a). These results imply significant differences in their metabolites among controls, GD and GO patients. Then volcano plot analysis was applied to identify potential differential metabolites accounting for the differentiation (Fig. 2b). Metabolites with VIP values > 1, *p* < 0.05 were regarded as significantly different. Based on our criteria, a total of 348 metabolites exhibited significant differences between the GO and control groups, 392 metabolites were significantly differentially expressed between the GD and control groups, and 298 metabolites differed significantly between the GD and GO groups. In more detail, compared with the control group, 213 metabolites were up-regulated and 135 metabolites were down-regulated in the GO group, while 234 metabolites were up-regulated and 158 metabolites were down-regulated in the GD group. Comparing with the GD group, there were 155 up-regulated metabolites levels alongside 143 down-regulated metabolite levels in the GO group (Supplementary Table 1). These results revealed that the serum displayed unique metabolic responses according to GD and GO. Furthermore, we performed Kyoto Encyclopedia of Genes and Genomes (KEGG) enrichment analysis on the significantly altered metabolites. The differential enriched pathways in GO and GD group included tyrosine metabolism, Trp metabolism, neurotransmitter and receptor metabolism, glycerophospholipid metabolism and synthesis of non-unsaturated fatty acids. Notably, the Trp metabolic pathway was presented in the differential enrichment of both GO and GD groups (Fig. 2c). Based on this, we further analyzed the levels of L-Trp and Trp metabolites detected by our metabolomics in three groups. Our metabolomics profiling demonstrated no significant difference in the levels of L-Trp among three groups, however, the results revealed a remarkable decrement in the levels of IAA and ILA in the GD cohort, as well as a notable reduction in IAA levels in the GO group compared to the control group (Fig. 2d). It is a pity that IPA was not detected in our metabolomics profiling. These findings suggest that GD and GO might be closely related with Trp metabolites rather than Trp.

### Serum Trp metabolites altered in GO patients

**Fig. 3:**
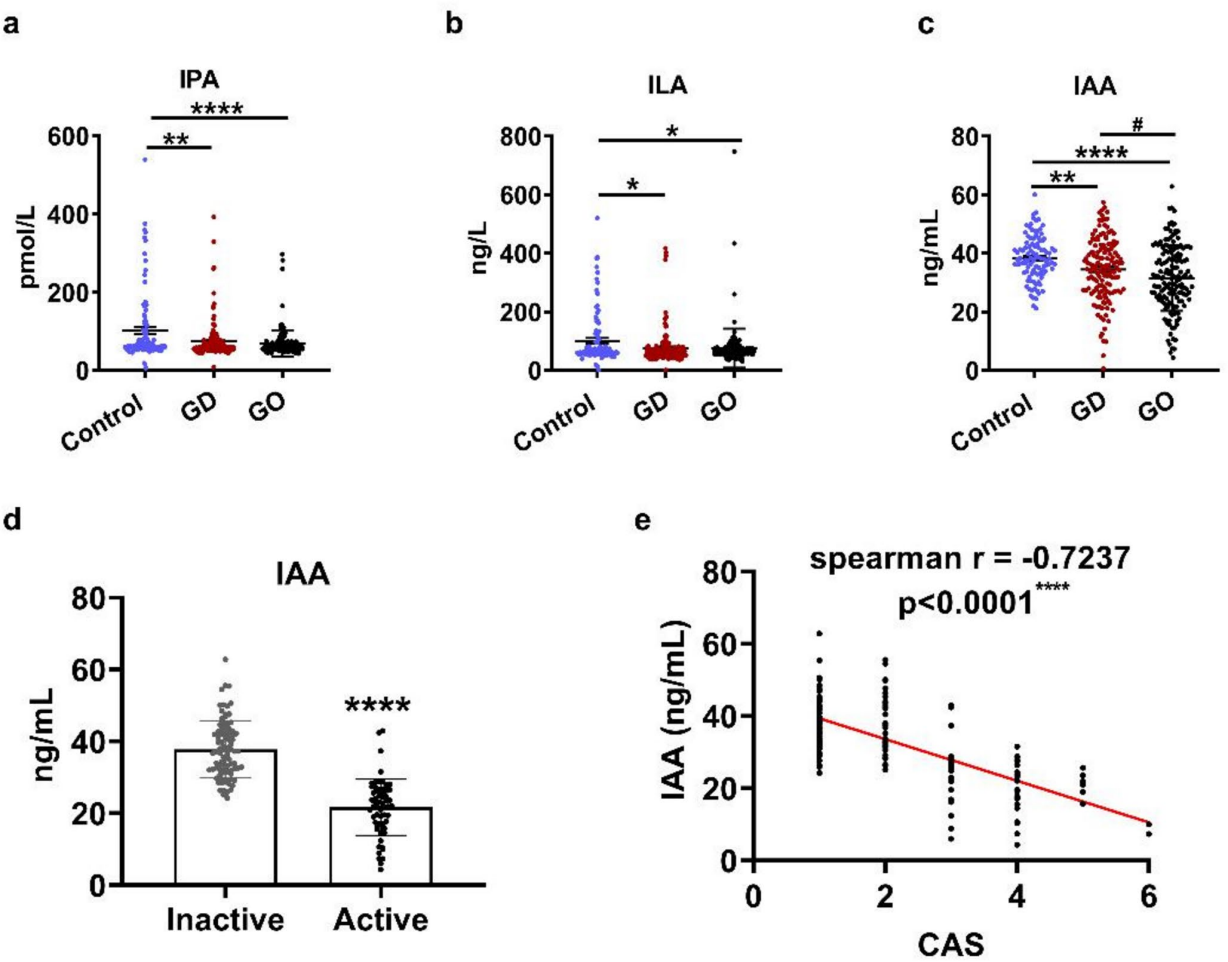
Circulating IPA, ILA and IAA were reduced in patients with GO. **a-c** 145 GD patients, 156 GO patients, and 100 healthy volunteers were enrolled in this study. Serum levels of IPA (**a**) ILA (**b**) and IAA (**c**) were examined by EILSA. Data were expressed as the mean ± SD. *P < 0.05, **P < 0.01, ***P < 0.001, ****P < 0.0001 vs. Control; ^##^*P* < 0.01 vs. GD. Control: Healthy volunteers; GD: Graves’ disease; GO: Graves’ orbitopathy. IPA: indolepropionic acid; ILA: indole-3-lactate; IAA: indoleacetic acid. (**d)** Comparison of serum IAA concentrations between active (*n* = 62) and inactive (*n* = 94) GO patients. Data were expressed as the mean ± SD. ****P < 0.0001 vs. Inactive. (**e)**, Spearman correlation analysis between serum IAA concentrations and CAS in GO patients (*n* = 156). *P* < 0.05 had considered statistically significance. CAS: clinical activity score

Given our discovery that Trp metabolites were closely related to GO and GD, we hypothesized that those metabolites may potentially serve as biomarkers for these diseases. To address this issue and to further confirm the results of our serum metabolomics, serum levels of IPA, ILA and IAA were measured in GD patients, GO patients and healthy volunteers (Control) by ELISA. Gender and age differed significantly in GO patients compared with GD group and Control. The levels of FT3, FT4 and TRAb in GD and GO patients were significantly higher as compared to the Control, while the level of TSH was notably decreased in the two groups. No significant difference was detected in the FT3, FT4, TSH and TRAb between GD and GO group. The above-mentioned clinical characteristics were presented in Table 1. Interestingly, ELISA assay results showed that, compared with controls, the serum levels of IPA, ILA and IAA were markedly lower in both GD and GO patients (Fig. 3a-c). Notably, when comparing with GD patients, the serum level of IAA was markedly lower in GO patients (Fig. 3c). These results suggested that IAA is the most promising candidate as a novel biomarker for GO. Therefore, to investigate the potential of IAA as a biomarker for the progression of GO, we classified GO patients into inactive (*n* = 94) and active (*n* = 62) groups based on the CAS value (inactive and active groups with a cut-off point of 3, respectively defined as CAS < 3 for inactive and CAS ≥ 3 for active GO). No significant difference was detected in the gender, age, FT3, FT4 and TSH between the two groups. While the levels of TRAb in active GO group were significantly higher as compared to the inactive group. The above-mentioned clinical characteristics were presented in Table 2. The results demonstrated that the serum levels of IAA were significantly lower in active GO patients than that in inactive ones (Fig. 3d). Given that CAS is mostly used index for assessing GO activity, the Spearman correlation analysis was performed to explore whether serum IAA levels correlate with CAS. Interestingly, serum levels of IAA were negatively correlated with CAS in GO patients (Fig. 3e).

**Table 1:**
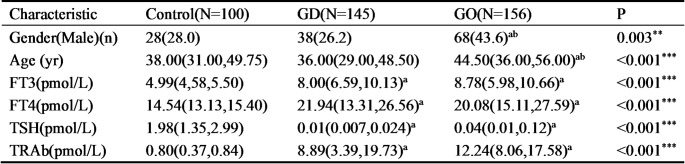
Characteristics of the participants at baseline

Values were n (%), Median (IQR). IQR: interquartile range; Control: Healthy volunteers; GD: Graves’ disease; GO: Graves’ orbitopathy; FT3: Free Triiodothyronine; FT4: Free Thyroxine; TSH: Thyroid-stimulating hormone; TRAb: thyrotropin Receptor Antibodies. ^**^P < 0.01, ^***^P < 0.001. Values with superscript letters: ^a ^significantly different when compare with Control; ^b^ significantly different when compare with GD

**Table 2:**
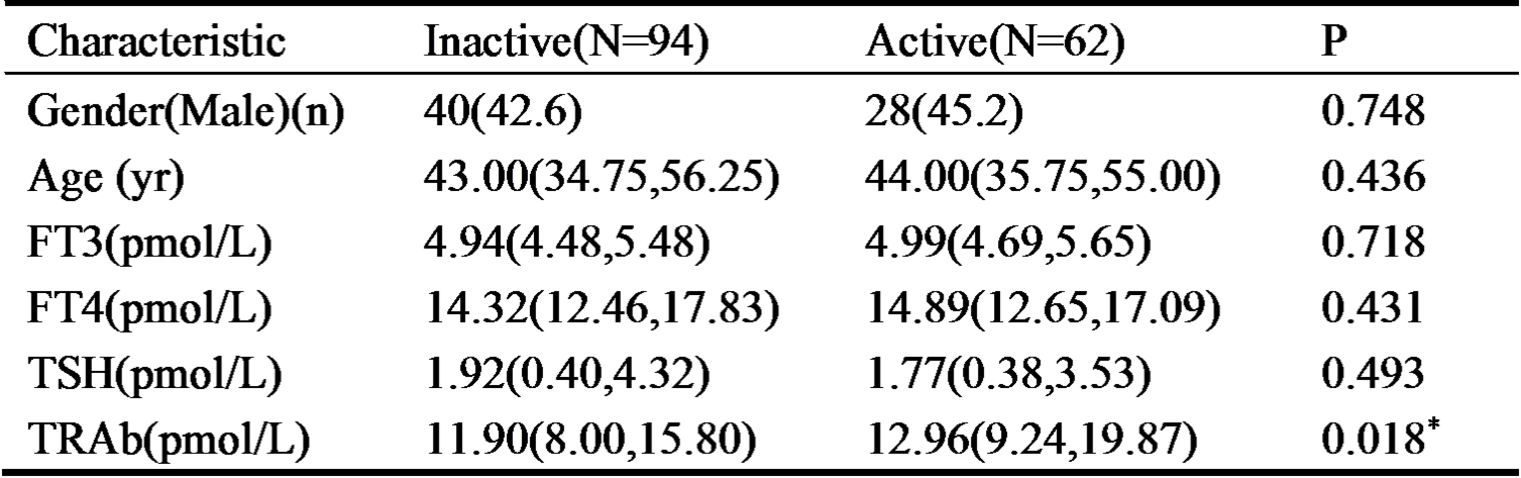
Characteristics of GO patients at baseline

Values were n (%), Median (IQR). IQR: interquartile range; GO: Graves’ orbitopathy; FT3: Free Triiodothyronine; FT4: Free Thyroxine; TSH: Thyroid-stimulating hormone; TRAb: thyrotropin Receptor Antibodies. **P < 0.01

It is well known that serum TRAb levels correlate with GO activity and severity. To explore whether Trp metabolites correlate with TRAb, the regression analysis was performed in our study. Considering the significant differences in age, gender and levels of TRAb among the three groups of patients (*P* < 0.001), the multivariate linear regression was performed. Each Trp metabolite was used as a dependent variable, while TRAb as an independent variable, with adjustments made for age and gender. The results showed that the concentrations of IAA and IPA exhibited a negative correlation with TRAb after adjustment for age and gender (*P* < 0.05) (Table 3). These findings further suggested that Trp metabolites, especially IAA may be promising biomarkers for GO progression.

**Table 3:**
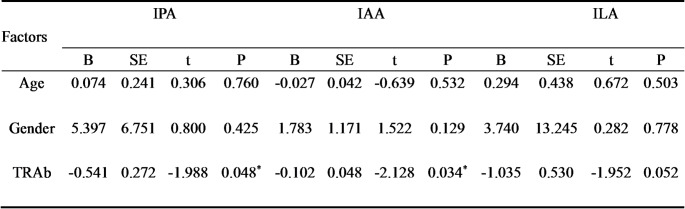
Multiple linear regression analysis of factors affecting trp metabolites in GO

GO: Graves’ orbitopathy; Trp: tryptophan; IPA: Indole-3-propionate; IAA: Indoleacetic acid; ILA: Indole-3-lactate; TRAb: Thyrotropin Receptor Antibodies. Note: ^*^P < 0.05, ^***^P < 0.001

### Trp metabolites improved TNFα-induced OFs proliferation and inflammation

Given that the IPA, ILA and IAA serum levels were significantly down-regulated in patients with GD and GO, we speculated that these metabolites might serve as important therapeutic targets for GO. To test this hypothesis, we conducted a series of in vitro experiments using primary cultured human OFs. Previous studies have confirmed that there is no significant difference in cell morphology and function of OFs between GO patients and healthy controls after in vitro subculture of retrobulbar connective tissue [27]. In the present study, we revealed that there was also no significant difference in proliferation and inflammation of OFs between GO patients and healthy controls after in vitro subculture of retrobulbar connective tissue (Suppl Fig. 1a-c). Therefore, the OFs derived from retrobulbar connective tissue of healthy individuals were used in subsequent experiments in this study.

**Fig. 4:**
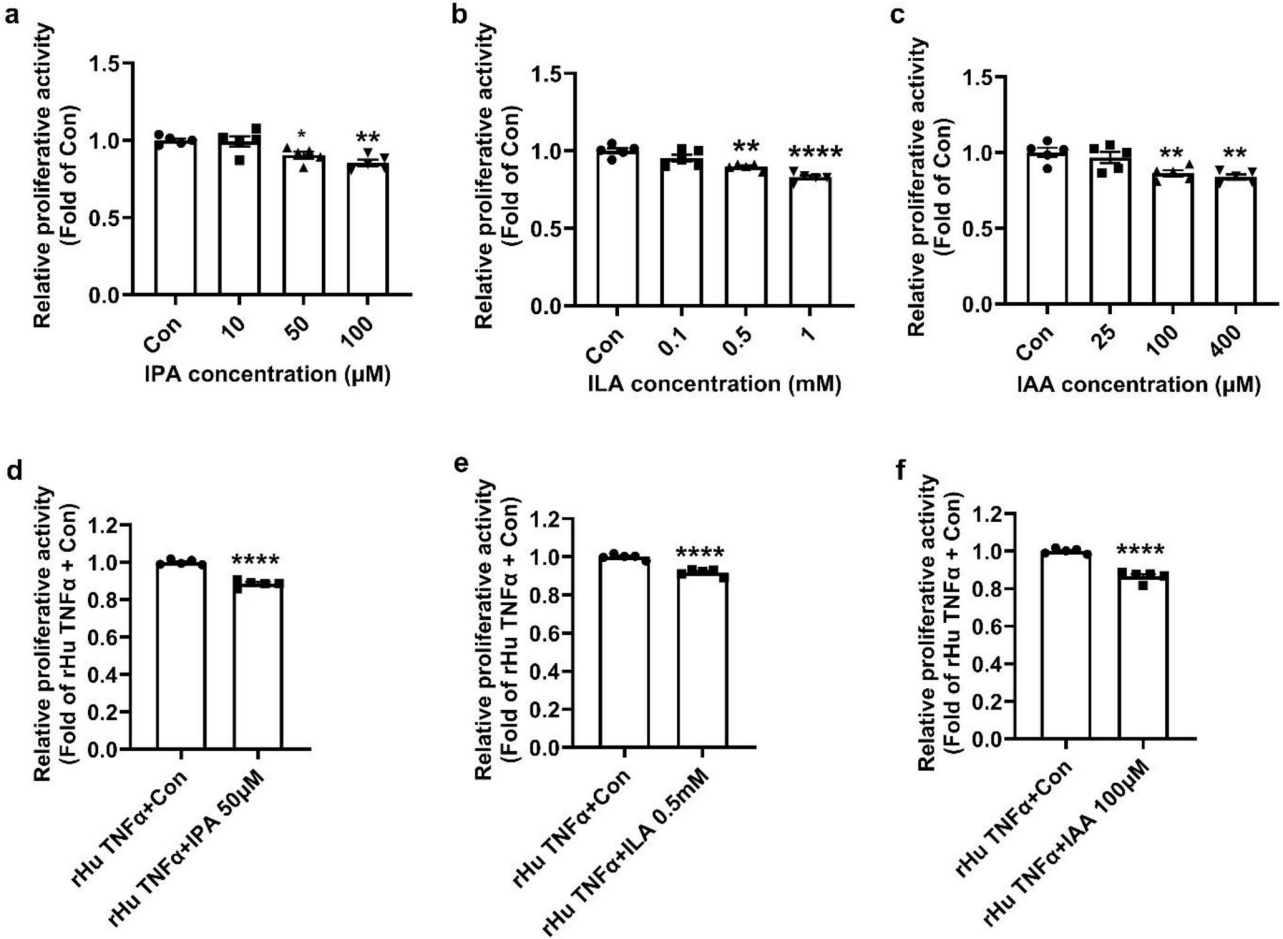
IPA, ILA and IAA improved TNFα-induced proliferation in human OFs. **a-c** Human OFs were treated with different concentrations of the 3 Trp metabolites or the equivalent amount of solvent (DMSO) for 24 hours (h) as described in the Materials and Methods section. The proliferation activity of the OFs was assessed via CCK-8 assay. The effects of IPA (**a**), ILA (**b**) and IAA (**c**) on proliferation activity were displayed as the relative cell viability. All these 3 Trp metabolites exhibited a dose-dependent decrease in cell proliferation activity when compared with control within the selected working concentration range. *n* = 5. Data were presented as mean ± SEM. *P < 0.05, **P < 0.01, ****P < 0.0001 vs. Con cells. **d-f** Human OFs were treated with 20ng/ml rHu TNFα or the equivalent amount of solvent, along with IPA (50µM), ILA (0.5 mM) and IAA (100µM) or the equivalent amount of solvent at the same time for 24 h. The proliferation activity of the OFs was assessed via CCK-8 assay. The effects of IPA (**d**), ILA (**e**) and IAA (**f**), on TNFα induced OFs proliferation were displayed as the relative cell viability. *n* = 5. Data were presented as mean ± SEM. ****P < 0.0001 vs. rHu TNFα + Con cells

**Fig. 5:**
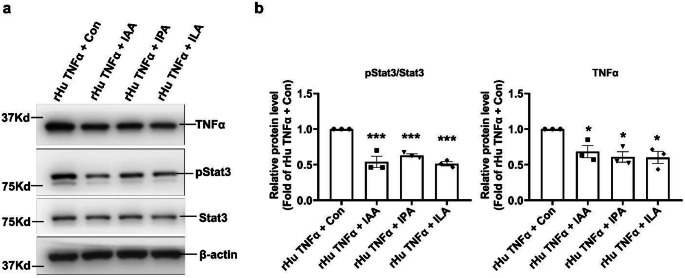
IPA, ILA and IAA improved TNFα-induced inflammation in human OFs. **a-b** Human OFs were treated with 20ng/ml rHu TNFα or the equivalent amount of solvent, along with IAA (100 µM), IPA (50µM) or ILA (0.5 mM) or the equivalent amount of solvent at the same time for 24 h. The inflammation levels of the OFs were assessed via WB assay. IAA, IPA and ILA significantly alleviated rHu TNFα stimulated pStat3 and TNFα levels. Representative images were shown in (**a**) and the quantitative data were shown in (**b**). *n* = 3. Data were presented as mean ± SEM. *P < 0.05, **P < 0.01, ***P < 0.001 vs. rHu TNFα + Con cells

Considering that cell proliferation and inflammation are important features of OFs in patients with GO, we initially evaluated the effects of these 3 Trp metabolites on cell proliferation using CCK-8 assay. The results showed a dose-dependent decrease in cell proliferation activity in cells treated with IPA, ILA and IAA compared with control group (Fig. 4a-c). TNFα, which is upregulated in OFs, plays a crucial role of inducing proliferation and proinflammatory cytokines in GO [3]. In line with the literature, further experiments showed that rHu TNFα could significantly induce cell proliferation in OFs (Suppl Fig. 2). It is worth noting that IPA, ILA and IAA could remarkably improve rHu TNFα induced OFs proliferation (Fig. 4d-f). Subsequently, the results of WB assays showed that rHu TNFα could significantly induce inflammation in OFs, as evidenced by significantly increased levels of pStat3 and TNFα (Suppl Fig. 3). Interestingly, IPA, ILA and IAA remarkably alleviated rHu TNFα induced OFs inflammation (Fig. 5a-b). These findings indicated that IPA, ILA and IAA may represent important therapeutic targets for GD and GO by ameliorating OFs inflammation and proliferation.

### Trp metabolites blocked TNFα-induced activation of the Akt signaling pathway in human OFs

**Fig. 6:**
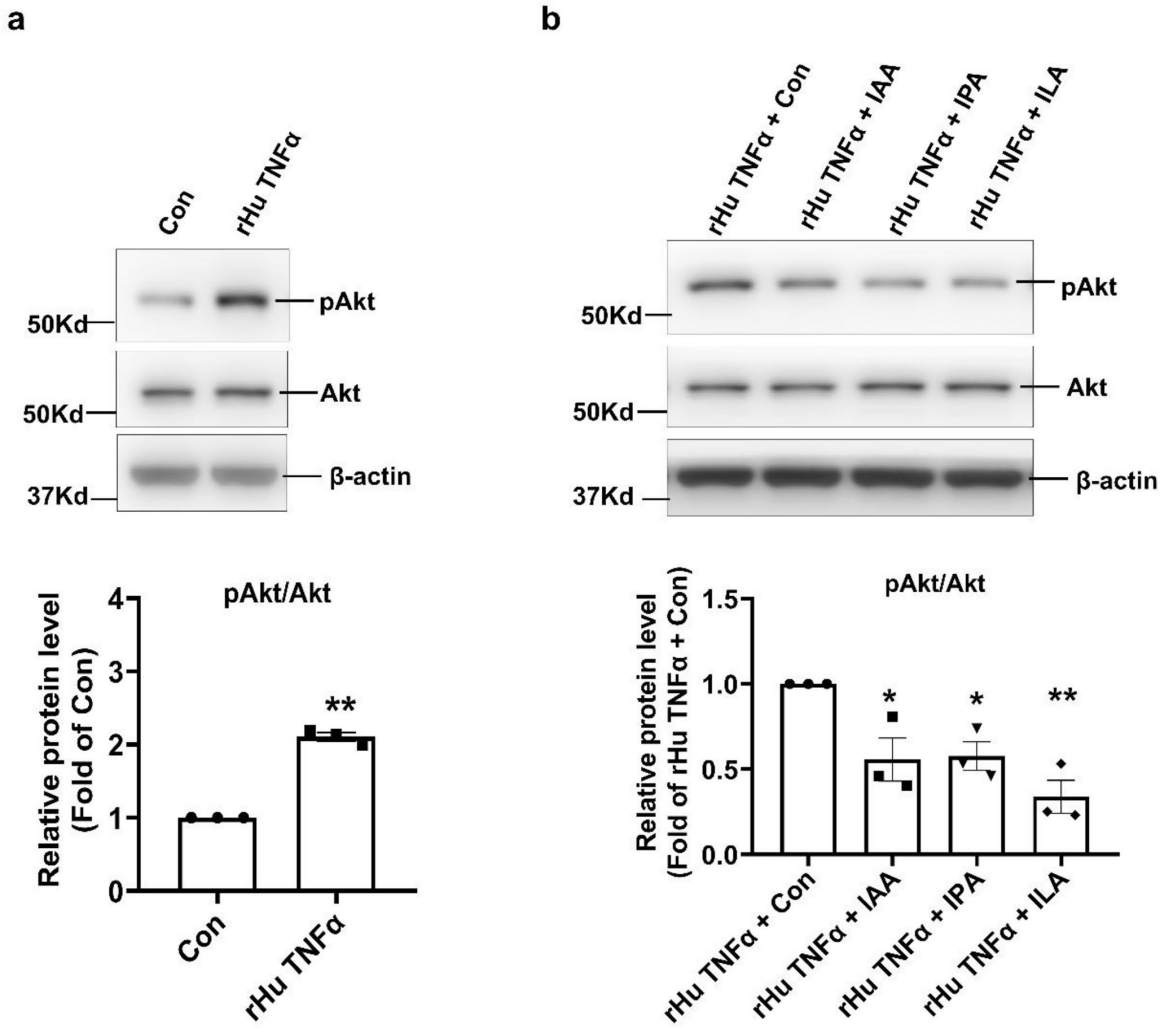
IPA, ILA and IAA blocked TNFα-induced activation of the Akt signaling pathway in human OFs. **a** Human OFs were treated with 20ng/ml rHu TNFα or the equivalent amount of solvent for 24 h. The Akt phosphorylation levels of the OFs were assessed via WB assay. TNFα intervention could significantly increase the protein level of pAkt. Representative images were shown in the up and the quantitative data were shown in the below. *n* = 3. Data were presented as mean ± SEM. **P < 0.01 vs. Con cells. **b** Human OFs were treated with 20ng/ml rHu TNFα or the equivalent amount of solvent, along with IPA (50µM), ILA (0.5 mM) or IAA (100µM) or the equivalent amount of solvent at the same time for 24 h. The Akt phosphorylation levels of the OFs were assessed via WB assay. IPA, ILA and IAA significantly blocked rHu TNFα stimulated pAkt levels. Representative images were shown in the up and the quantitative data were shown in the below. *n* = 3. Data were presented as mean ± SEM. *P < 0.05, **P < 0.01 vs. rHu TNFα + Con cells

Akt signaling pathway has been verified to play a role in GO adipogenesis, cell migration, cell proliferation and inflammation in several studies, and these studies pointed to phosphorylation of Akt at serine 473 (phospho-Akt, pAkt) was elevated in OFs in GO, which was considered to be an important pathogenesis of GO [28–32]. Trp metabolites IAA was reported to inhibited the PI3K/Akt/mTOR pathway, thereby suppressing differentiation and extracellular matrix deposition in lung fibroblasts [33]. Therefore, to investigate the potential mechanism for Trp metabolites IPA, ILA and IAA in improving inflammation-induced dysfunction in OFs, we investigated their effects on the Akt signaling pathway. We were interested to find that TNFα treatment significantly promoted the activation of the Akt pathway, as evidenced by increased pAkt (Fig. 6a), and IPA, ILA or IAA treatment was all able to block Akt phosphorylation induced by TNFα (Fig. 6b). This finding suggested that Akt signaling pathway plays an important role in the protective effect of IPA, ILA and IAA against inflammation-induced dysfunction in OFs in GO.

## Discussion

GO is a thyroid-associated autoimmune disease that is vision-threatening and cosmetically damaging [1]. It occurs in approximately 30–50% of GD patients. Corneal scarring caused by exposure or optic nerve compression can result in vision loss and, in severe cases, blindness, significantly affecting the life quality of patients [34]. Numerous studies suggested a bidirectional communication system between the thyroid and the gut. Furthermore, intestinal flora has a significant and immediate impact on Trp metabolites and thus on human immunity. So far, the role of Trp metabolites in GO patients has not been elucidated. The present study started with intestinal microbia sequencing analysis to identify differences in Trp associated flora, followed by an exploration of the serum Trp metabolomics profile. To further confirm the change in serum, we examined levels of Trp metabolites by ELISA and analyzed their correlation with clinical indexes including CAS and TRAb. Furthermore, we elucidated the potential mechanisms underlying anti-inflammation and anti-proliferation effects of Trp metabolites in GO.

More and more studies have demonstrated the relationship between microbiota composition and the thyroid disease in recent years [35]. Hui-Xian Yan et al. had provided evidence that patients with GD undergo significant changes in intestinal microbiota compared with healthy controls [36]. Our previous study showed the changed intestinal microbiota in GO patients. Intriguingly, another study performed in European countries reported that the *Firmicutes* to *Bacteroidetes* ratio was significantly higher in GD/GO than in healthy controls, and they also defined a microbiome signature and identified changes associated with autoimmunity as distinct from those due to hyperthyroidism [37]. In the current study, we further reanalyzed the change of flora in GO patients. At the genus level, the relative abundances of 35 genera in GO patients were significantly different compared to controls. Furthermore, remarkable differences in Trp associated genera were observed between the two groups at different levels. At the phylum level, abundance of *p_Firmicutes* in GO group were down-regulated significantly. At the genus level, abundance of *g_Anaerostipes* in GO group were decreased markedly compared with controls. Evidence showed that *Firmicutes* and *g_Anaerostipes* play a crucial role in boosting the Trp metabolism during the degradation of dietary fiber in the body, thereby protecting the intestinal barrier. Our study firstly demonstrated a significant down-regulation of specific genera associated with Trp metabolism in GO patients.

Trp metabolites generated by the gut microbiota contribute to intestinal and systemic homeostasis by regulating immune cells. These metabolites, such as IAA, IA and ILA, affect immune responses through the AhR [38, 39]. Evidence showed that Trp metabolites and metabolic enzymes were significantly different between healthy individuals and patients with Inflammatory Bowel Diseases [40]. Oral IAA administration can modulate reactive oxygen species (ROS)-degrading enzymes such as glutathione peroxidase 3, thereby decreasing the accumulation of ROS in cancer cells [41]. Xiumei Yan et al. demonstrated that Fructooligosaccharides ameliorates allergic symptoms and impacts Th17/Treg balance in mice by modulating Trp metabolites including IAA [42]. Additionally, IPA levels are lower in blood samples from individuals with type 2 diabetes as compared to the lean controls [43]. Based on this, we expanded the sample size to further verify the differences in IAA, ILA and IPA among the healthy control group, the GD group and the GO group via ELISA assay. The serum levels of IPA, ILA and IAA were markedly down-regulated in GD and GO patients compared with controls. Moreover, the serum level of IAA was markedly lower in GO patients compared to GD patients. Notably, active GO patients had significantly lower IAA levels compared to inactive ones. Moreover, the levels of IAA were negatively correlated with CAS and serum TRAb. These findings further elaborated that Trp metabolites including IAA, ILA and IPA might play an important role in GD and GO disease, furthermore, IAA may serve as the most promising biomarker for GO progression. The examination of serum IAA may help early identifying active GO patients.

GO is characterized by orbital inflammatory infiltration and activation of OFs, which mediate proliferation, adipogenesis, hyaluronan production, and myofibroblast differentiation [44]. Previous studies support the antifibrotic and anti-inflammatory properties of IAA. Krishnan et al. provided evidence that IAA attenuated inflammatory responses under lipid loading and reduced the expression of fatty acid synthase expression in hepatocytes [45]. Evidence showed that treatment of OFs with TNFα induces inflammation and differentiation into myofibroblasts [46]. To explore the protective role of Trp metabolites in the OFs, we investigated their interaction with OFs in context of TNFα induced proliferation and inflammation. Our data showed that Trp metabolites remarkably inhibited proliferation, and the expression of the inflammation marker pStat3 in OFs. Here, for the first time, we provided evidence that IAA, ILA and IPA may play a protective role against proliferation and inflammation in GO patients.

The P13K/Akt signal pathway exerts an essential role in OFs affecting GO adipogenesis. In orbital fibroblasts from GO patients, 69% of patients demonstrated stimulated p-Akt [47]. Previous reports demonstrated that Akt was activated partly by upregulating PPAR-γ in the OFs from GO patients [32]. The pretreatment of Akt inhibitor can significantly decrease the hyaluronic acid concentration in OFs [48]. Jingxue Sun et al. have showed evidence that plasma exosomes deliver mir-885-3p and can inhibit the Akt signaling pathway to improve the glucocorticoid sensitivity of OFs [49]. In order to investigate the potential mechanism for Trp metabolites IPA, ILA and IAA in improving TNFα-induced inflammation and proliferation in OFs, we investigated their effects on the Akt signaling pathway. In our study, IPA, ILA or IAA treatment could down-regulate Akt phosphorylation induced by TNFα in OFs. Our results complemented the evidence that GO may benefit from blockade of Akt signaling pathways.

There were several limitations in the present study. First, we focused exclusively on the role of Trp metabolites in OFs in GO, without considering the potential crosstalk between Trp metabolites and immunocytes. Second, although the current study demonstrated different intestinal bacteria and microbiota-derived Trp metabolites profile in GO, the flora transplantation in animal model should be carried out to confirm the protective role of Trp metabolites and elucidate the underlying mechanisms. Finally, because it is difficult to enroll GD and GO patients who did not receive anti-hyperthyroidism therapy in the department, we cannot exclude the effect of methimazole on the levels of Trp metabolites in patients. In the future, we will try to enroll more newly diagnosed GD and GO patients in multicenter to further explore the change of Trp metabolites.

## Conclusions

In conclusion, our study firstly defined changes of microbiota-derived Trp metabolites in GO, furthermore, demonstrated that IPA, ILA and IAA exert potential therapeutic activity in GO by ameliorating inflammation and controlling proliferation in OFs. Microbiota-derived Trp metabolites may serve as potential therapeutic targets to the treatment of GO. Thus, maintaining gut microbiota production of IAA, ILA and IPA, for example, by dietary intervention regimes or oral probiotic supplement, may exert beneficial effects on GO management.

## Electronic supplementary material

Below is the link to the electronic supplementary material.

Supplementary Material 1

Supplementary Material 2

## Data Availability

The data that support the findings of this study are available from the corresponding author upon reasonable request.
