# Supplementary material for: Tryptophan metabolites exert potential therapeutic activity in graves’ orbitopathy by ameliorating orbital fibroblasts inflammation and proliferation
Source: J Endocrinol Invest. 2025 May 27;48(8):1781–95. doi: 10.1007/s40618-025-02593-6 (PMC12313747; doi:10.1007/s40618-025-02593-6)
Supplement: Supplementary file 2 — Supplementary Material 2 [file 40618_2025_2593_MOESM2_ESM.docx]

**Suppl Figures and figure legends**


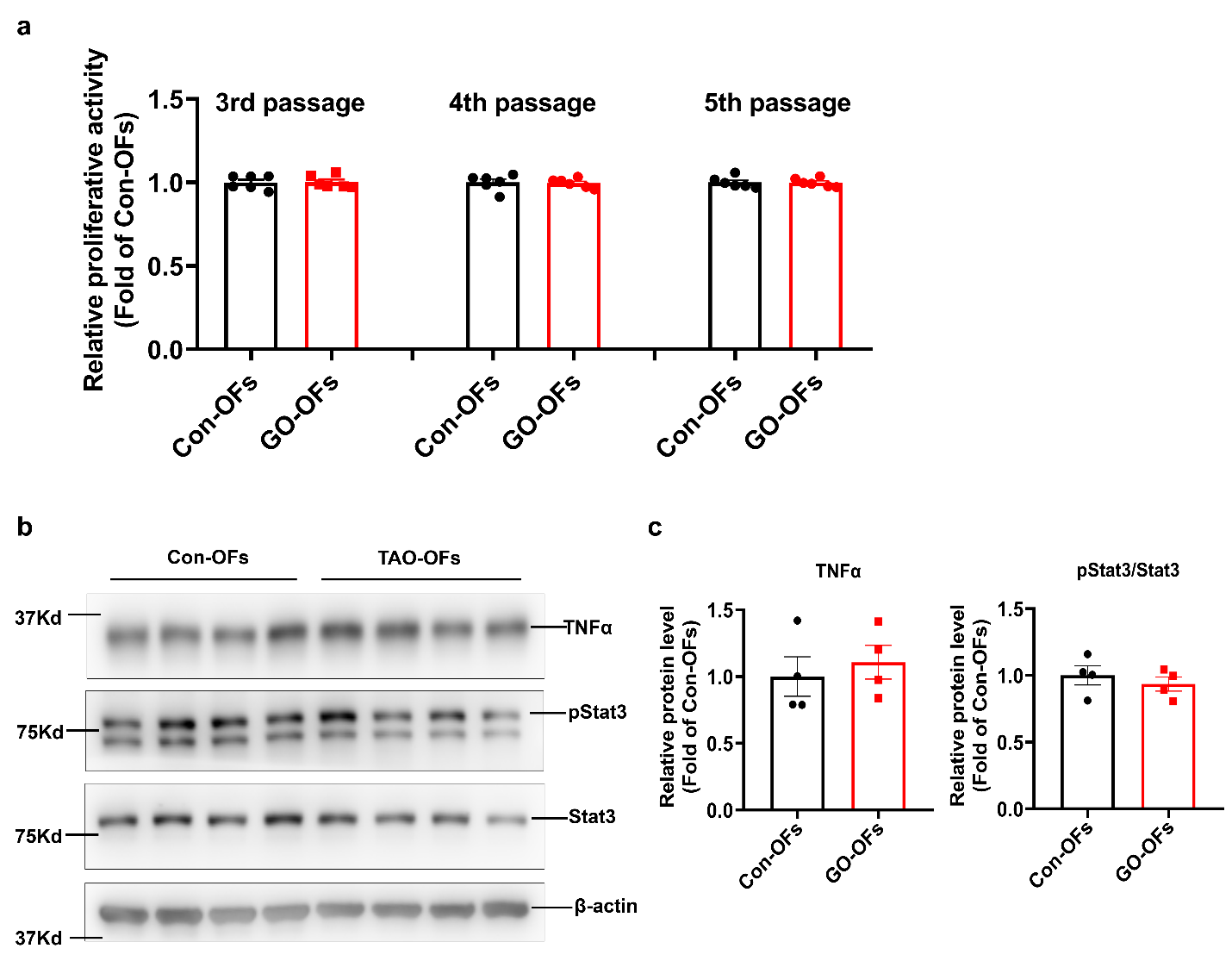


**Suppl Fig. 1 OFs from either control individuals or patients with GO revealed no differences in proliferation and inflammation**

**a,** OFs from both control individuals and patients with GO at different passage were seeded at the same density and allowed to adhere overnight. The proliferation activity of the OFs was assessed via CCK-8 assay. n = 6. Data are presented as mean ± SEM. Con-OFs: OFs from both control people; GO-OFs: OFs from patients with GO.

**b-c,** The 3rd passage of OFs from both control individuals and patients with GO were seeded at the same density and allowed to adhere overnight, and the inflammation levels of the OFs were assessed via WB assay. Representative images were shown in (**b**); Quantitative data are shown in (**c**). n = 4. Data are presented as mean ± SEM.


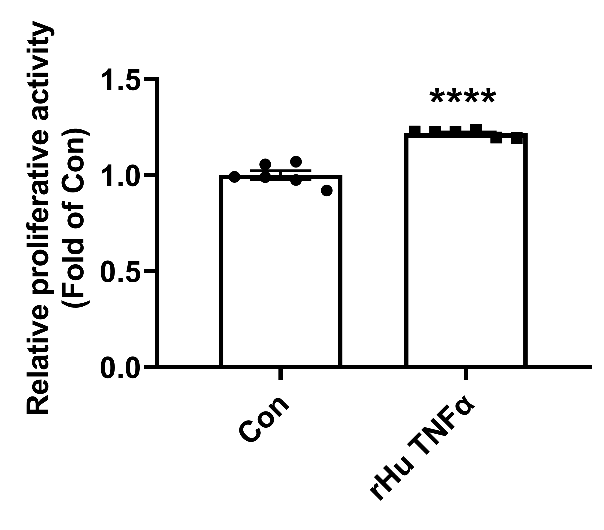


**Suppl Fig. 2 TNFα markedly induced cell proliferation in human OFs**

Human OFs were treated with 20ng/ml rHu TNFα or the equivalent amount of solvent for 24 h, and the proliferation activity of the OFs was assessed using CCK-8 assay. n = 6. Data are presented as mean ± SEM. *****P* < 0.0001 vs. Con cells.


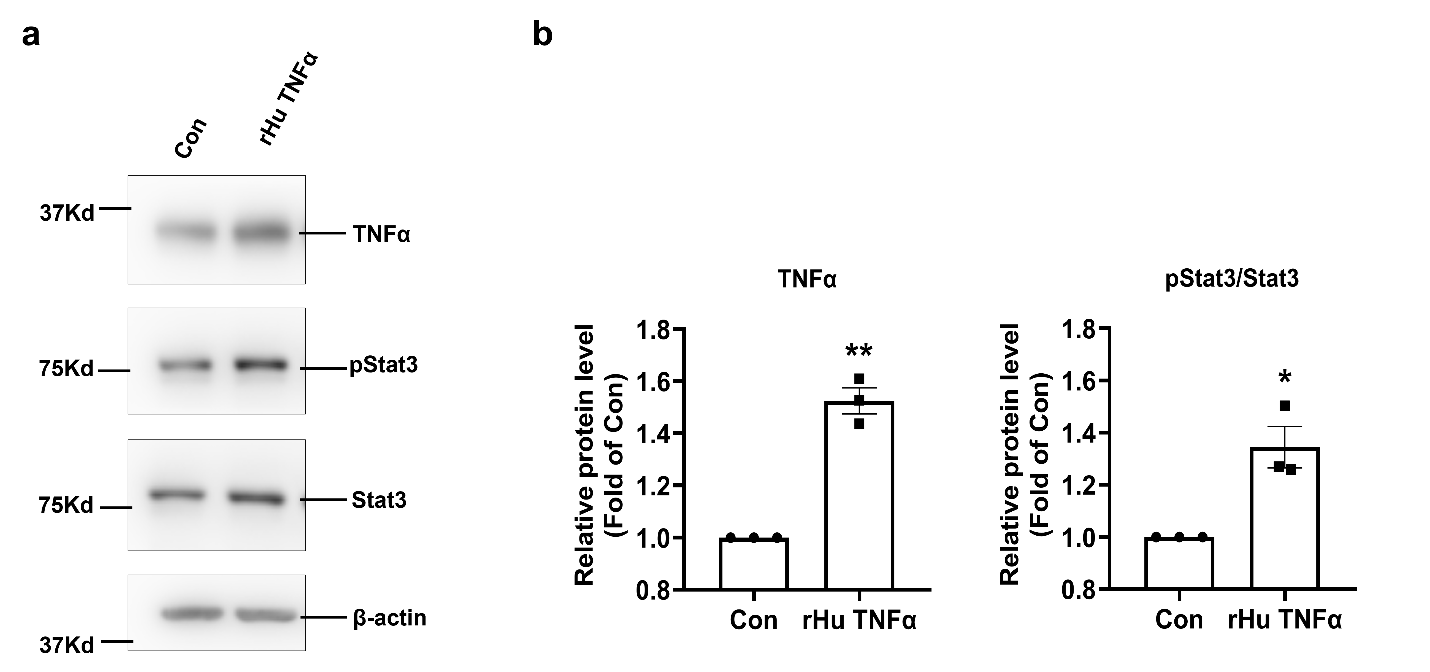


**Suppl Fig. 3 TNFα markedly induced inflammation in human OFs**

**a-b,** Human OFs were treated with 20ng/ml rHu TNFα or the equivalent amount of solvent for 24 h, and the inflammation levels of the OFs was assessed via WB assay. Representative images were shown in (**a**); Quantitative data are shown in (**b**). n = 3. Data are presented as mean ± SEM. **P* < 0.05, ***P* < 0.01 vs. Con cells.
